# Colocutaneous Fistula Formation Following Inguinal Hernia Repair: A Case Series

**DOI:** 10.7759/cureus.59842

**Published:** 2024-05-07

**Authors:** Nikolaos Koliakos, Andrianos-Serafeim Tzortzis, Dimitrios Papakonstantinou, Anargyros Bakopoulos, Nikolaos Pararas, Evangelos Misiakos, Emmanouil Pikoulis

**Affiliations:** 1 Department of Abdominal Surgery, Erasme Hospital, Free University of Brussels (ULB), Brussels, BEL; 2 Department of Ear, Nose & Throat (ENT), Lister Hospital, East and North Hertfordshire NHS Trust, Stevenage, GBR; 3 Third Department of Surgery, Attikon University General Hospital/National and Kapodistrian University of Athens, School of Medicine, Athens, GRC

**Keywords:** hernia mesh, laparoscopic tapp repair, enterocutaneous fistula, emergency surgery, hernia

## Abstract

Mesh placement remains the standard of care for inguinal hernioplasty, whether through the classic open approach or the transabdominal preperitoneal (TAPP) approach. Though both techniques are generally safe, they can occasionally result in visceral injuries, albeit infrequently. Mesh migration into the intestines is a morbid situation requiring emergency treatment. We present two male patients who developed mesh-enterocutaneous fistula several years after inguinal hernia repair. The first patient with a history of a bilateral TAPP hernia repair was admitted to the emergency department and underwent bilateral complete mesh removal, limited right colectomy, and wedge resection of the sigmoid colon, due to mesh erosion. The second patient, with a history of a left inguinal hernia treated by open mesh repair, presented to the emergency department complaining of intense pain in his left inguinal area. Erosion of the prosthetic mesh into the sigmoid and a colo-cutaneous fistula was identified, with sigmoidectomy and en bloc excision of the adherent mesh and end-colostomy being performed.

Mesh erosion into the intestinal tract is a rare but serious condition. In patients presenting with a subcutaneous abscess in the inguinal region, clinicians should maintain a high level of suspicion for intrabdominal inflammation arising from mesh erosion into adjacent viscera. Surgical management becomes necessary in symptomatic cases or instances of fistulization.

## Introduction

Mesh placement is the gold standard technique for inguinal hernioplasty. One of the rarest complications encountered is mesh-related erosion into adjacent abdominal viscera. This situation may be encountered when the mesh is exposed to the contents of the abdominal cavity due to the lack of a peritoneal interface. This occurs when the mesh becomes exposed to the abdominal cavity contents due to inadequate closure of the peritoneal defect during transabdominal preperitoneal (TAPP) hernia repair or gradual penetration of the mesh through the inguinal canal's back wall [1]. Symptomatic cases necessitate surgical intervention to remove the affected mesh and address any associated visceral damage [2].

In this paper, we present two rare cases of an 82-year-old and an 83-year-old patient with mesh-related intestinal erosions, resulting in colocutaneous fistula formation.

## Case presentation

Case 1

**Figure 1:**
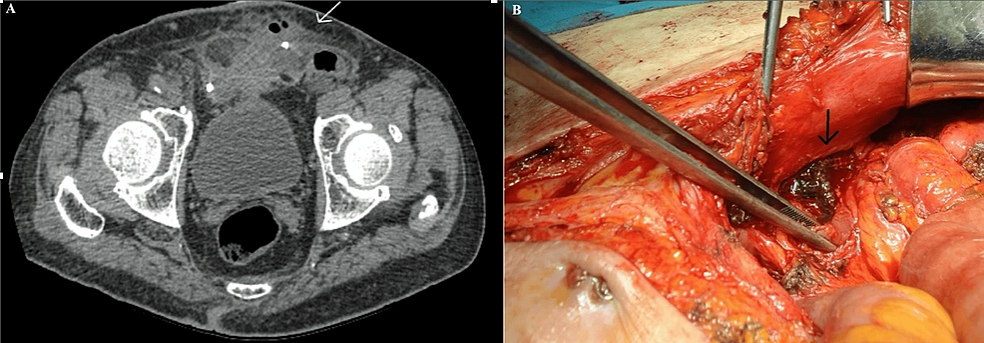
(A) Mesh fixating material into an abdominal wall fluid collection with air bubbles (white arrow). (B) Intraoperative photograph indicating intestinal erosion due to mesh migration and a fistulous tract on the left lower portion of the anterior abdominal wall (black arrow).

An 82-year-old male patient presented to the emergency department due to a green-colored discharge from a sinus tract in the region between the umbilicus and the pubic tubercle. He reported low-intensity lower abdominal pain for the past two months. Intestinal transit was preserved. His medical history included atrial fibrillation and hyperlipidemia, and he had undergone mitral valve replacement and laparoscopic bilateral inguinal hernia repair using the TAPP procedure two years prior. Physical examination did not reveal any other notable findings. Laboratory tests upon admission showed normal inflammatory marker levels but marked hypoalbuminemia (1.9 g/dL). Contrast-enhanced abdominal computer tomography indicated communication between the ileum and the skin in the region of TAPP repair, with gastrografin detected within the sinus tract (Figure 1A).

The length of the fistula was 4 cm until the abdominal wall surface. Hence, erosion of mesh prosthetic material into the gastrointestinal tract was suspected as a cause of the enterocutaneous fistula. Considering the patient's compromised nutritional status, conservative management was initially chosen. This approach involved initiating parenteral nutrition along with high-protein enteral nutrition to address the patient's nutritional needs.

The symptoms persisted and 10 days later the patient underwent a diagnostic laparoscopy. During laparoscopy, the terminal ileum and the sigmoid colon were found to be strongly adhered to the hypogastric peritoneum. Immediate conversion to open laparotomy was decided via midline incision. The terminal ileum and the polypropylene mesh used for the right inguinal hernia repair had evolved into a mass in direct contact with the abdominal wall fistula (Figure 1B). The mesh was carefully dissected from the spermatic structures and was removed. Due to an extended bowel injury, a formal right colectomy was performed. After a thorough examination of the left inguinal area, mesh migration into the sigmoid colon was documented. Wedge sigmoid colectomy and en bloc excision of the adherent mesh were performed. Gastrointestinal continuity was achieved by an end-to-end hand-sewn anastomosis. The peritoneal defects were approximated with sutures. However, no effort was made to repair the inguinal defects ensuing after mesh removal.

Histopathologic examination of the surgical specimen revealed erosion and ischemic lesions of the examined bowel, without any signs of malignancy. Postoperatively, the patient developed a surgical site infection that was treated with drainage and daily changes with saline-soaked cotton gauze dressings. No other complications were encountered, and the patient was discharged on the ninth postoperative day. There has been no evidence of hernia recurrence after a two-year follow-up period with the patient being in good health ever since.

Case 2

**Figure 2:**
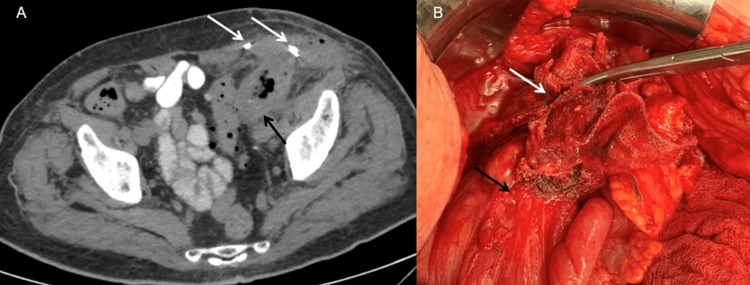
(A) Abdominal CT scan reveals an abdominal abscess communicating with the sigmoid colon (black arrow) and mesh fixating materials identified in the left inguinal area. (B) Intraoperative photograph indicating sigmoid colon erosion (black arrow) by left inguinal mesh (white arrow).

An 83-year-old male patient presented to the emergency department complaining of intense pain in his left inguinal area. His past medical history included atrial fibrillation, burn injuries in his abdomen and the lower extremities, sigmoid diverticular disease, and left inguinal hernia treated by classic open hernia repair with mesh insertion 20 years ago. Physical examination revealed scarring from the previous burn injuries and a tender mass accompanied by skin edema and inflammation in the left lower abdominal quadrant. Laboratory tests at the time of admission revealed a rise in the inflammatory markers (white cell count 19, reference range 3.6-11.0 x10^9/L; CRP 16, reference range 0.0-0.7 mg/dL). Contrast-enhanced abdominal computer tomography showed sigmoid colon diverticulosis, an abscess in contact with the sigmoid colon, and mesh fixating materials located in the left lower abdominal wall (Figure 2A).

As a result, erosion of the prosthetic mesh into the sigmoid was suspected as the cause of this presentation. Percutaneous drainage was performed, followed by two weeks of conservative treatment, which consisted of antibiotics and daily wound care. At first, the discharge during drainage was purulent, but after four days the content was mainly fecal, and a colocutaneous fistula was suspected and later confirmed by CT fistulography.

The patient was taken into theatre, due to failure of the conservative approach, and open laparotomy via midline incision was performed. The mesh was carefully dissected from the adjacent anatomic structures of the abdominal wall and the retroperitoneum. The bowel injury due to the mesh erosion into the sigmoid was quite extended (Figure 2B). As a result, sigmoidectomy along with en-bloc excision of the adherent mesh and end-colostomy were performed. The peritoneal defects were approximated with interrupted sutures and the inguinal hernia was primarily repaired.

The postoperative period was uneventful and the patient was discharged on the eighth postoperative day. Histopathologic examination of the excised specimen confirmed erosion and ischemic lesions of the examined bowel. In follow-up, there was no hernia recurrence four months later.

## Discussion

Elective hernia repair is one of the most frequent surgical procedures performed and is indicated regardless of age [3]. Mesh hernia repair has prevailed as the most popular hernia repair technique. Amongst its rarest complications is the migration of the prosthetic mesh into nearby viscera, causing obstruction or perforation [4]. The TAPP procedure has been linked to a small but significantly higher percentage of visceral injuries relative to other types of hernia repair surgery [1].

Intestinal erosion symptoms can vary, ranging from mild vague abdominal pain to a disastrous enterocutaneous fistula [1]. Postoperative fistula may be recognized even one year after the initial operation. Increasing pain, swelling, and tenderness around the incision is a very common clinical presentation. In the first case, the fistula was the result of erosion of the terminal ileum and sigmoid colon due to polypropylene mesh migration occurring late in the postoperative course. Lo et al. were the first to publish a case of such complication, presenting as small bowel obstruction [5], whereas Karls et al. presented a case of an inflammatory mass containing the mesh, the sigmoid colon, and a loop of small bowel [6]. Therefore, this condition may present with a variety of symptoms and for this reason, a high index of suspicion is required to enable a timely diagnosis.

Mesh-related fistulae have recently emerged as a rare postoperative complication. There is limited data to support any pathogenic mechanisms of this entity. According to Agrawal and Avill, both chronic contact between viscera and prosthesis, and foreign body reaction due to polypropylene mesh placement remain the main factors contributing to fistulization [7]. The role of diverticulosis in the fistula formation remains unclear. There are cases in which diverticular disease coexisted with mesh-related fistulae, although no direct implication could be noted [7-11]. However, the presence of diverticula is speculated to be a risk factor [9]. In the second case, the mesh had eroded through the wall of the sigmoid colon eventually leading to the formation of a colocutaneous fistula, requiring open laparotomy with sigmoidectomy and en bloc excision of the adherent mesh. In such cases, where mesh erosion coexists with diverticular disease, diagnostic dilemmas may arise, nevertheless, surgery effectively addresses both the colocutaneous fistula and the required excision of the inflammatory mass containing the mesh [8-12]. 

Enterocutaneous mesh-related fistula complicating inguinal hernia repair is rare and may follow a long period of latency. Careful handling of tissues and ensuring adequate coverage of the prosthetic mesh material with a peritoneal interface during hernia repair surgery is very important in avoiding this morbid complication that almost uniformly requires surgery for resolution.

## Conclusions

In patients presenting with a subcutaneous abscess in the inguinal region, a high level of suspicion should be maintained for intrabdominal inflammation stemming from mesh erosion into adjacent viscera. Surgical intervention becomes imperative in symptomatic cases or instances of fistulization.
